# Regarding ‘efficacy analysis of combined nerve combing and decompression for occipital neuralgia based on the classification of neurovascular relationships’

**DOI:** 10.1080/07853890.2026.2719985

**Published:** 2026-08-19

**Authors:** Chul Bum Cho

**Affiliations:** Department of Neurosurgery, St. Vincent’s Hospital, College of Medicine, The Catholic University of Korea, Seoul, Republic of Korea

Dear Editor

We read with interest the report by Zhang et al. [1], who proposed a four-subtype classification of greater occipital nerve (GON) compression by the occipital artery and presented favorable outcomes after a combined combing-and-decompression procedure. Anatomical observations were carefully documented, and the surgical details were valuable. However, the entire framework and the clinical claim built upon it rests on a causal premise that the available anatomical and histological evidence does not support that contact between the occipital artery and the GON is itself a cause of occipital neuralgia (ON). We raise this point not to dispute that some patients improve but to question what is being treated.

The central difficulty is the specificity. The authors cited an ON incidence of approximately 3.2 per 100,000. However, arterial contact with the GON is not a rare finding, as it is close to a normal anatomical state. In the cadaveric series of Janis et al. the GON and occipital artery intersected in 54% of dissected sides of pain-free donors, ranging from single crossing to helical intertwining [2]. More pointedly, Shimizu et al. set out to test the analogy that motivates the present classification—whether the occipital artery compresses the GON in a manner analogous to vascular compression of the trigeminal root—and found that the two structures crossed in every specimen, with an indentation of the nerve by the artery present in all of them [3]. A morphological feature observed in essentially every asymptomatic individual cannot, on its own, account for a disorder that affects a few people per 100,000. According to the logic of the proposed classification, almost the entire population is anatomically destined for ON.

Histology is still more damaging to the causal interpretation. Shimizu et al. examined GON at the points of arterial indentation and found no evidence of mechanical nerve damage, even in specimens with atherosclerosis of the occipital artery, and explicitly concluded that their study did not provide direct evidence that the occipital artery contributes to ON at the contact point [3]. Likewise, subsequent studies failed to identify occipital artery pathology as a mechanism underlying ON-related headaches [4]. The intracranial neurovascular-conflict paradigm invoked here does not transfer to this setting; the trigeminal and facial nerves are vulnerable at the central-to-peripheral myelin transition (the Obersteiner–Redlich zone), where a pulsatile vessel can provoke ephaptic transmission. The GON in the nuchal subcutaneous plane is a wholly peripheral, Schwann cell-myelinated nerve with no such transition zone, and the external carotid–derived occipital artery does not reproduce the fixation and pressure conditions of a posterior-fossa arterial loop. Borrowing the ‘neurovascular conflict,’ vocabulary therefore imports a mechanism whose anatomical prerequisite is absent.

A further confound concerns about what the operation does. In every case, we resected a segment of the occipital artery and stripped the epineurium. This is not decompression in the microvascular sense—mobilizing and interposing material to spare both nerves and vessels, but rather selective devascularization combined with partial epineural denervation. A single-arm before-and-after design cannot distinguish relief obtained by removing a culprit vessel from the non-specific effect of partial denervation of the natural history of a fluctuating pain disorder or of an unblinded, self-reported outcome. Notably, the authors’ own data argue against the vascular hypothesis: the parallel-contact subtype (Type D), in which the artery runs alongside the nerve without true compression, had by far the poorest result—one-third of patients unimproved—and accounted for all four recurrences. If pulsatile arterial contact was the operative cause, resection should have helped most of these patients. The cohort is also difficult to interpret as ON specific, since 73.6% had comorbid chronic migraine and inclusion required a positive anesthetic block, which enriched responders before any surgery was performed.

However, occipital nerve pain can have a genuine compressive substrate. Space-occupying lesions, such as a nerve-sheath tumor or lipoma, and entrapment by hypertrophied fascia or muscle mechanisms, the authors and others have documented [5]—produce a fixed, demonstrable, and anatomically specific compression that is conceptually distinct from incidental contact with a normal pulsating artery. We would suggest that the proposed subtypes be presented as a useful descriptive map of intraoperative surgical anatomy rather than as an etiological classification, and that the causal claim awaits the evidence that would actually support it: a comparison of arterial-contact prevalence between symptomatic and asymptomatic sides (or patients and controls), histological correlation of contact with demyelination, and, ultimately, a controlled trial with a credible sham arm. Until then, ‘decompressing’ a structure found in nearly everyone risks attributing to the artery a benefit that may owe more to denervation and to the regression of a naturally remitting symptom.

We thank the authors for their thought-provoking contributions and opportunity to comment.

## Data Availability

No new data were generated or analyzed in support of this letter. Data sharing is not applicable to this article, as no datasets were created or analyzed.
